# Medical Association of Georgia

**Published:** 1866-05

**Authors:** 


*Medical Association of Georgia.*—It seems the appointment for the next meeting of this body has not yet been made, but we suppose it will not be longer delayed than is absolutely necessary to have a full understanding among the officers, committees, etc., in regard to time, place, etc. It is certainly determined to have the meeting some time the present year, and we shall heartily concur in any arrangement that may be thought advisable. Let the appointment for the meeting be made, so that the organization does not pass away by default, and we are content.

Those who feel inclined to write prize essays, may do so with the assurance, we are informed, that the prizes will be ready for the successful competitors.

The following are the officers elected and committees appointed at the last meeting of the Association, held in Atlanta, April 10, 1861:

Dr. J. T. Banks, of Griffin, President.

Dr. J. F. Alexander, of Atlanta, 1st Vice President.

Dr. V. H. Taliaferro, of Columbus, 2d Vice President.

Dr. A. G. Thomas, (then) of Atlanta, Secretary, &c.

Committee appointed to raise $100, to be distributed as prizes for the three best essays presented to the next meeting—$50 for the first, $30 for the second, and $20 for the third. Committee: Drs. O'Keefe, Taliaferro and Boyd.

Committee to examine prize essays—Drs. H. Coe, A. Means, J. F. Alexander, H. W. Brown and T. C. H. Wilson.

Committee appointed to procure orator and alternate for next meeting—Drs. Coe, Dannelly and Brown.

On motion, the President was required to appoint, at his convenience, a committee of arrangements.

*Resolved*, That the Committee on Prize Essays be instructed, in awarding prizes, to give preference to subjects of practical interest to the profession, and to award the prizes publicly, during the session of the Association, in cash or its equivalent, at the discretion of the committee.

